# Unveiling the viroporin arsenal in plant viruses: Implications for the future

**DOI:** 10.1371/journal.ppat.1012473

**Published:** 2024-09-05

**Authors:** Guanwei Wu, Jianping Chen, Aiming Wang, Fei Yan

**Affiliations:** 1 State Key Laboratory for Managing Biotic and Chemical Threats to the Quality and Safety of Agroproducts, Institute of Plant Virology, Ningbo University, Ningbo, China; 2 Key Laboratory of Biotechnology in Plant Protection of MARA and Zhejiang Provincial Key Laboratory of Green Plant Protection, Institute of Plant Virology, Ningbo University, Ningbo, China; 3 London Research and Development Centre, Agriculture and Agri-Food Canada, London, Ontario, Canada; Shanghai Center for Plant Stress Biology, CHINA

## Abstract

Viroporins are small, hydrophobic viral proteins that modify cellular membranes to form tiny pores for influx of ions and small molecules. Previously, viroporins were identified exclusively in vertebrate viruses. Recent studies have shown that both plant-infecting positive-sense single-stranded (+ss) and negative-sense single-stranded (-ss) RNA viruses also encode functional viroporins. These seminal discoveries not only advance our understanding of the distribution and evolution of viroporins, but also open up a new field of plant virus research.

## What are viroporins and what do they do?

The concept of viroporins originated from early observations indicating that cells infected with influenza virus A (IAV) exhibited heightened permeability to ions and small molecules. Amantadine was one of the first antiviral drugs used to treat IAV in the 1960s, yet it was not until the mid-1980s when the target of its therapeutic action was identified to be the M2 protein of IAV. Further research in 1992 demonstrated that M2-mediated ion channel activity is sensitive to altered external pH induced by amantadine [1]. This pivotal discovery accelerated the recognition of viroporins and the identification of various examples from human/animal RNA and DNA viruses, such as the protein 2B (P2B) from picornavirus, the p7 protein from hepatitis C virus, the envelope (E) protein from severe acute respiratory syndrome coronavirus (SARS-CoV), and the XP protein from human astrovirus [2–4].

Viroporins identified from these vertebrate viruses are usually small and highly hydrophobic and vary in size from 50 to 120 amino acid residues. These proteins are capable of self-oligomerization and forming aqueous channels in the membranes of host cells. This pore-forming ability is a common strategy used by both viruses and bacteria for pathogenesis and serves various functions, including signaling and defense [5,6]. Viroporins are categorized into 3 classes, based on their membrane-spanning domains: those with a single transmembrane domain, those with 2 domains that form a helix-turn-helix hairpin motif, and those with more than 2 transmembrane domains [7]. Given their capacity to alter membrane permeability, viroporins have been demonstrated to play critical functions throughout the viral infection cycle. These include facilitating viral entry, participating in the replication of viral genome, aiding in the assembly of virus particles, and promoting the entry into and release from host cells [8]. Some viruses, such as SARS-CoV, even encode more than 1 viroporin, including the E, ORF3a, and ORF8b, which can selectively conduct protons across cellular membranes, leading to the loss of organelle acidification [2]. Furthermore, certain viroporins, such as P2B, polyprotein P2BC, and P3A from poliovirus or the E from coronavirus, are known to induce intracellular membrane rearrangements, creating viroplasms-new membrane vesicles that act as sites for viral replication [9]. Additionally, viroporins have the ability to perturb intracellular calcium homeostasis by influencing the influx of extracellular calcium and/or causing leakage of calcium from intracellular stores, such as the mitochondria, endoplasmic reticulum (ER), and Golgi apparatus [10]. This disruption can trigger a cascade of cellular signaling and immune responses [11]. The diverse roles of viroporins highlight their importance in viral pathogenesis and emphasize the necessity for further investigation, especially regarding their presence and functions in plant viruses, which remains poorly understood.

## What is known about viroporins in plant viruses?

**Fig 1:**
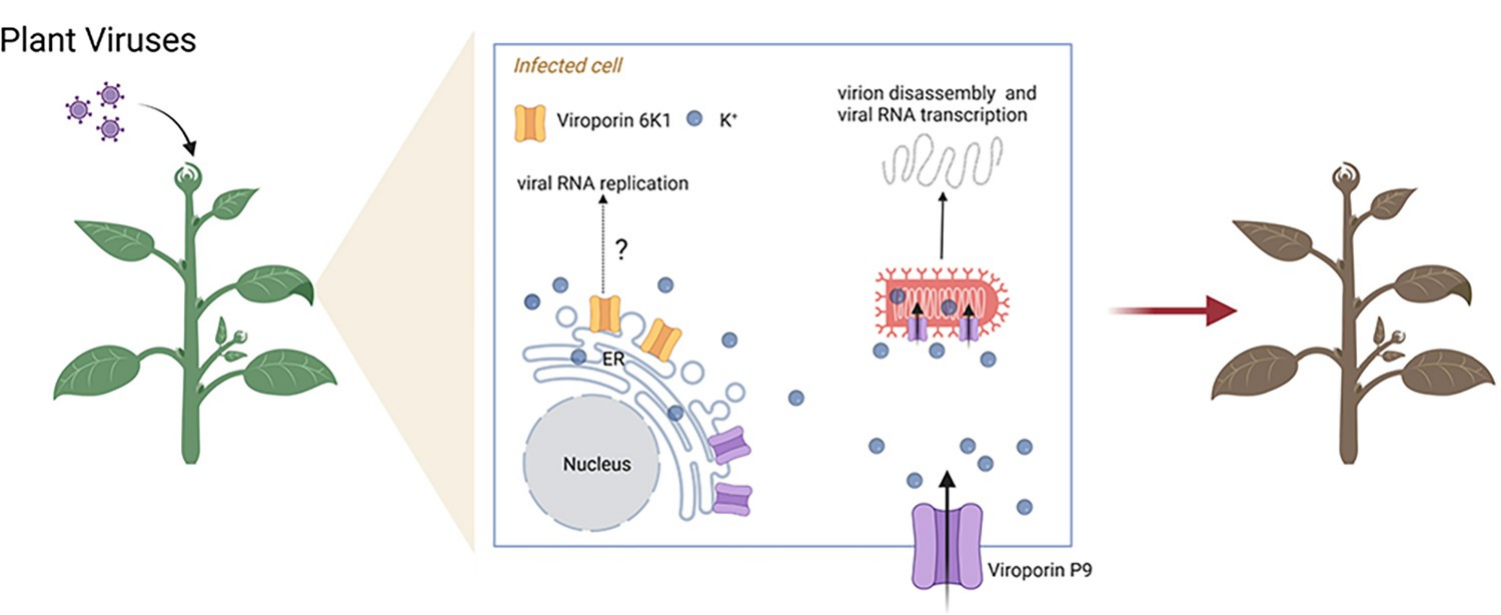
Schematic representation of viroporins encoded by plant viruses impacting viral infection in plants. During the course of plant virus infections, viruses exploit their own viroporins to facilitate ion exchange, a strategy that aids in establishing a successful viral infection and advancing the development of disease symptoms. The precise roles of the ER-localized viroporins 6K1 encoded by +ssRNA potyviruses in this intricate process remain to be comprehensively explored. The viroporin P9 encoded by −ssRNA cytorhabdoviruses facilitates K^+^ uptake across the PM, thereby increasing K^+^ content within plant cells. Moreover, P9 assembles into tiny viroporin structures that trigger virion disassembly and initiate viral RNA transcription. Created with BioRender.com.

The brome mosaic virus (BMV; genus *Bromovirus*, family Bromoviridae) RNA replication protein 1a has been demonstrated to exhibit viroporin activity previously, allowing it to permeabilize ER membranes in yeast [12], despite lack of a classic viroporin structure. Recently, Chai and colleagues experimentally proved the presence of the first typical plant virus-encoded viroporin, namely the six-kilodalton peptide 1 (6K1) from (+ssRNA) turnip mosaic virus (TuMV; genus *Potyvirus*, family Potyviridae) [13]. Potyviridae stands as the largest family of plant-infecting RNA viruses, harboring numerous economically significant pathogens [14]. Employing Alphafold2, Chai and colleagues [13] predicted the structure of 6K1, revealing a hairpin-like helix-turn-helix folding pattern in the monomeric form of 6K1. Through protein–protein interaction assays and size exclusion chromatography, they observed 6K1 is able to oligomerize into pentamers, with a predicted funnel-shaped central channel. Subsequent membrane permeabilization assays, using wild-type 6K1 and a pentamer-disrupting mutant, confirmed 6K1 is capable of inducing plasma membrane (PM) permeabilization. Moreover, ion conduction assays in yeast demonstrated 6K1 can transport potassium (K^+^). These findings establish 6K1 as a functional viroporin. Viral infection assays further revealed the crucial role of 6K1’s viroporin activity in virus replication and symptom development (Fig 1). Additionally, the authors observed viroporin activity in 6K1 or 7K (equivalent to 6K1) proteins from other potyvirids, and these proteins possess a conserved secondary structure, featuring a hydrophobic α-helix at their N-terminus, along with critical amino acid residues—specifically valine at position 19 and phenylalanine at position 23—located on the inner surface of the putative tunnel [13]. Almost simultaneously, a separate study from another group reported that the P9 protein from (−ssRNA) barley yellow striate mosaic virus (BYSMV; genus *Cytorhabdovirus*, family Rhabdoviridae) also displayed viroporin activity [15]. BYSMV P9 forms oligomers and localizes to the ER, viral envelope, and PM [15]. Moreover, P9 exhibits K^+^ channel activity, which aids in the virion disassembly and the release of genomic RNA for mRNA transcription (Fig 1). Low K^+^ levels inhibit BYSMV infection, and BYSMV P9 can enhance K^+^ uptake under K^+^-deficient conditions. Consistently, a point mutant in P9 (P19^G14T^) lacking K^+^ channel activity severely impairs the infection of barely plants [15]. The P9 in plant cytorhabdoviruses share a viroporin-like sequence feature, including a single central transmembrane domain, an aromatic-residue-rich N-terminal domain, and a highly basic C terminus [15]. These original findings pave the way for further investigation into the existence and functions of viroporins in plant viruses.

## What is the role of identified plant viroporins in viral infection?

The prevalence of diseases caused by plant viruses calls for in-depth research into the molecular mechanisms underlying their pathogenesis. Among the least characterized of potyviral proteins is 6K1, whose function has been a subject of inquiry. Chai and colleagues’ research has shed light on the mechanism by which TuMV can directly utilize the viroporin activity of 6K1 to facilitate viral replication [13]. It is noteworthy that in addition to 6K1, TuMV encodes another six-kilodalton peptide, namely 6K2. While 6K2 is known for its role in the formation of vesicles where viral RNA, virus replicase, and host factors coexist, 6K1 from plum pox virus (PPV), a related potyvirus, has been observed to colocalize with 6K2-induced viral replication complexes (VRC) within infected cells [16], it is plausible that 6K2 could facilitate the targeting of 6K1 to VRC, thereby regulating the exchange of ions or small molecules across the VRC membranes. Further investigation is required to elucidate the specific organelles where 6K1 exerts its viroporin activity and to determine whether this activity is integral to VRC formation or the maintenance of ion homeostasis within these complexes. In a separate study, researchers demonstrated that the P9 protein of BYSMV forms viroporin structures and enhances K^+^ influx within the viroporin envelope. This influx, in turn, facilitates virion disassembly and the release of genome RNA, enabling the initiation of viral infection in plant cells [15]. Given that the P9 viroporin, while dispensable for virus infection in insect vectors, is strictly required for the transmission from insects to plants in the phloem, it presents an intriguing avenue of research to determine whether BYSMV P9 can exhibit viroporin activity within insect membranes. Finally, BMV 1a can act like a viroporin, permeabilizing the ER membranes to induce a more oxidized local environment, which in turn stimulates viral replication [12]. The mechanism underlying the cation conduction mediated by these viroporins and the broader biological implications of these findings remain to be fully explored.

## Do other plant viruses have the potential to encode viroporins?

As obligate intracellular parasites, plant viruses possess compact genomes encoding a limited number of proteins. In fact, many plant viruses encode at least 1 small hydrophobic protein with potential roles in viroporin activity. In additional to the 6K1 or 7K protein encoded by potyvirids [13], this includes the triple-gene-block protein 3 (TGBp3) from the family Alphaflexiviridae, the small cysteine-rich protein (CRP) encoded by members of the families Virgaviridae and Betaflexiviridae, the P6 protein from the family Closteroviridae, and the accessory genes in the *G*-*L* gene junctions of plant rhabdoviruses [17–20]. Moreover, recent reports have identified several small proteins encoded by the minus strand of positive-sense plant viruses [21,22]. Furthermore, evidence is mounting that geminiviruses, which have circular single-stranded DNA genomes, also encode small proteins with biological functions that may remain hidden [23,24]. Research has demonstrated that the CRP of potato mop-top virus from Virgaviridae can form multimers on endomembranes [25]. This ability is pivotal for virus–host interactions and is also a characteristic property of viroporins [2,26]. Thus, it is plausible that these viral-encoded small hydrophobic proteins may also function as viroporins within plant cells.

## Are host ion channels involved in plant viral pathogenesis?

In addition to viroporin-induced micro ion channels, viruses may also exploit host porins or ion channel proteins to facilitate their replication and spread. A prime example is the voltage-dependent anion channel (VDAC), found in the outer mitochondrial membrane, which controls metabolite flux, modulates the production of reactive oxygen species, and plays a role in mitochondrial-mediated apoptosis by releasing pro-apoptotic proteins [27,28]. VDAC is known for transporting crucial metabolites, including various ions such as K^+^, Na^+^, Cl^-^, Mg^2+^, and Ca^2+^. A recent study has revealed that TGBp1 of bamboo mosaic virus (BaMV; genus *Potexvirus*, family Alphaflexiviridae) interacts with *Nicotiana benthamiana* VDAC proteins, forming a complex that bolsters viral infection [29]. Moreover, the P1 protein of rice grassy stunt virus (RGSV; genus *Tenuivirus*, family Virgaviridae) has been reported to enhance viral infection by inhibiting K^+^ absorption in rice plants through modulating the activity of K^+^ channel protein AKT1 [30]. These examples highlight the potential for plant viruses to manipulate host ion channels and maintain ion homeostasis to facilitate their propagation. Therefore, ion channel blockers and drugs targeting viroporins could be an effective option in the fight against plant viruses.

In summary, the original findings that plant viruses also encode functional viroporins broadens our comprehension of viroporins beyond the traditional scope of human and animal viruses, promotes our understanding of distribution and evolutionary aspects of viroporins, and shed light on the role of viroporins and host ion channels in plant virus–host interactions.
